# Supplementary material for: Causal interplay between lactose intolerance and gut microbiota: a combined bidirectional Mendelian randomization and in vivo validation study
Source: Front Nutr. 2026 Jun 1;13:1803337. doi: 10.3389/fnut.2026.1803337 (PMC13265576; doi:10.3389/fnut.2026.1803337)
Supplement: Supplementary file 1 [file Data_Sheet_1.zip › supplementary materials/Forward/leave one out cross validation/ebi-a-GCST90027628.finngen_R12_E4_LACTONAS.pdf]

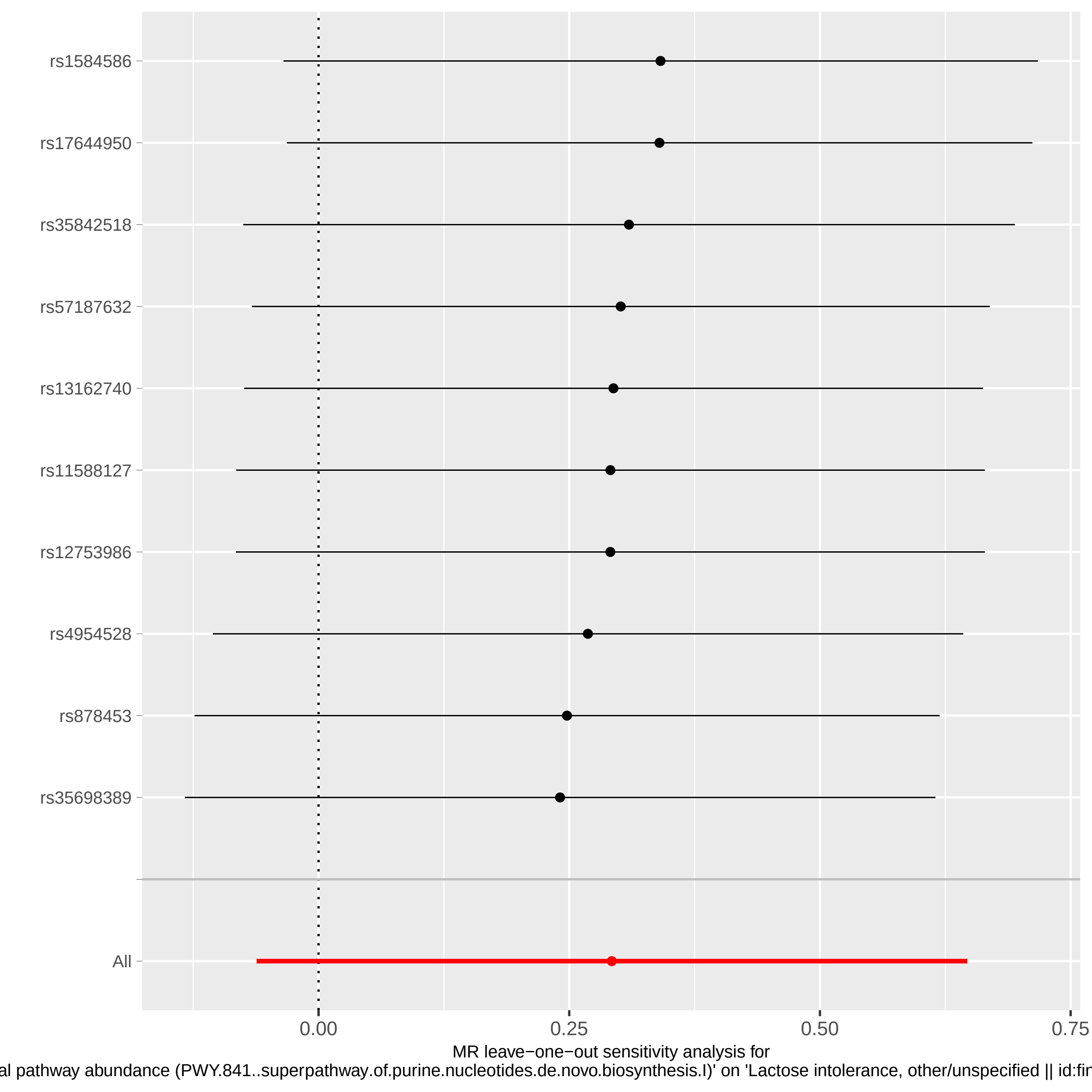
rs1584586
rs17644950
rs35842518
rs57187632
rs13162740
rs11588127
rs12753986
rs4954528
rs878453
rs35698389
All
0.00
0.25
0.50
0.75
MR leave−one−out sensitivity analysis for
al pathway abundance (PWY.841..superpathway.of.purine.nucleotides.de.novo.biosynthesis.I)' on 'Lactose intolerance, other/unspecified || id:fin
